# A Novel Phytogenic Formulation, EUBIO-BPSG, as a Promising One Health Approach to Replace Antibiotics and Promote Reproduction Performance in Laying Hens

**DOI:** 10.3390/bioengineering10030346

**Published:** 2023-03-10

**Authors:** Hieu Tran Nguyen Minh, Tien-Fen Kuo, Wen-Yu Lin, Tzu-Chia Peng, Greta Yang, Chih-Yu Lin, Ting-Hsiang Chang, Yu-Liang Yang, Cheng-Hsun Ho, Bor-Rung Ou, Chu-Wen Yang, Yu-Chuan Liang, Wen-Chin Yang

**Affiliations:** 1 Agricultural Biotechnology Research Center, Academia Sinica, Taipei 11529, Taiwan; minhhieutrannguyen94@gmail.com (H.T.N.M.); tienfen@gate.sinica.edu.tw (T.-F.K.); as0200509@gmail.com (T.-C.P.); greyang900204@gmail.com (G.Y.); zelda@gate.sinica.edu.tw (C.-Y.L.); uncaria8412@gmail.com (T.-H.C.); ylyang@gate.sinica.edu.tw (Y.-L.Y.); chho@gate.sinica.edu.tw (C.-H.H.); wcyang2023@gmail.com (W.-C.Y.); 2 Institute of Biotechnology, National Taiwan University, Taipei 11529, Taiwan; 3 Department of Animal Science and Biotechnology, Tunghai University, Taichung City 40704, Taiwan; bigeyesowl@gmail.com (W.-Y.L.); brou@thu.edu.tw (B.-R.O.); 4 Department of Microbiology, Soochow University, Taipei 11102, Taiwan; 5 Department of Life Sciences, National Taiwan Ocean University, Keelung 202301, Taiwan; 6 Graduate Institute of Integrated Medicine, China Medical University, Taichung City 406040, Taiwan; 7 Department of Life Sciences, National Chung Hsing University, Taichung City 40227, Taiwan

**Keywords:** antibiotic resistance genes (ARG), antibiotic resistance (AR), EUBIO-BPSG, gut microbiota, laying hens, short-chain fatty acids and derivatives (SCFA)

## Abstract

Gut microbiota play a key role in health maintenance and disease pathogenesis in animals. Dietary phytochemicals are crucial factors shaping gut bacteria. Here, we investigated the function and mechanism of a phytogenic formulation, EUBIO-BPSG (BP), in laying hens. We found that BP dose-dependently improved health and egg production in 54-week-old hens. Furthermore, BP was correlated with increased fecal *Lactobacillus*, decreased *Escherichia coli* and *Salmonella enterica*, and reduced antibiotic resistance (AR) and antibiotic resistance genes (ARG) in chicken stools. The 16S rDNA data showed that BP increased seven genera of probiotics and reduced 13 genera of pathogens in chicken feces. In vitro co-culture experiments showed that BP at 4 µg/mL and above promoted growth of *L. reuteri* while large 100- and 200-fold higher doses suppressed growth of *E. coli* and *S. enterica*, respectively. Mechanistic studies indicated that *L. reuteri* and its supernatants antagonized growth of *E. coli* and *S. enterica* but not vice-versa. Five short-chain fatty acids and derivatives (SCFA) produced from *L. reuteri* directly killed both pathogens via membrane destruction. Furthermore, BP inhibited conjugation and recombination of ARG via interference with conjugation machinery and integrase activity in *E. coli*. Collectively, this work suggests that BP promotes host health and reproductive performance in laying hens through regulation of gut microbiota through increasing probiotics and decreasing pathogens and spreading ARG.

## 1. Introduction

Gut microbiota modulate the function of the intestine and other organs in humans and animals [1]. In the digestive tract, gut bacteria participate in digestion, nutrient uptake, detoxification, and mucosal immunity [2]. They mediate health, growth performance, and reproductive performance in hosts. Gut pathogenic bacteria such as *Escherichia, Shigella, Salmonella, Streptococcus, Staphylococcus,* etc., cause diseases inside and outside the digestive system. In contrast, beneficial gut bacteria such as *Lactobacillus*, *Bifidobacterium*, *Bacteroides*, *Alistipes* [3], *Megamonas* [4], *Butyricicoccus* [5], *Megasphaera* [6], etc., contribute to host health, performance, and immunity via multiple mechanisms, including epithelial barrier function and adhesion, production of antibiotic metabolites, competitive inhibition of pathogenic bacteria, and immunity [7]. For instance, *Lactobacillus*, the most abundant gut probiotic, is beneficial for growth, reproduction, and host defense. The proportion of this genus decreases in aged animals [8]. Furthermore, *Lactobacillus* and other probiotics can produce antimicrobial metabolites that antagonize gut pathogens. Short chain fatty acids and derivatives (SCFA) such as acetic acid, lactic acid, propionic acid, 3-hydroxypropionic acid (3-HPA), and butyric acid are abundant in *L. reuteri* [9]. These suppress growth of pathogens such as *S. *enterica** and *E. coli* [10,11]. Accordingly, supplementation with exogenous probiotics and SCFA could improve host health and reproductive performance [12]. However, such supplementation with exogenous probiotics and their metabolites has sometimes resulted in controversial outcomes due to strain specificity, retention time in the animal gut, and the host status [13,14].

Imbalance of probiotics and pathogens, termed dysbiosis, in the gut leads to intestinal pathology, mental disorders, asthma, metabolic syndromes, autoimmune diseases, etc. Manipulation of gut microbiota may rectify gastrointestinal and extra-intestinal diseases. Host genetics, exogenous bacteria, diets, antibiotics, stress, and aging can shape the gastrointestinal bacterial community [15,16,17]. Based on their scientific and economical merits, laying hens are an interesting animal model for study of gut microbiota, host health, and reproduction. The global egg market was valued at USD 162.4 billion in 2019 [18], and 82.2 million tons yearly [19]. Laying hens establish their gut microbiota within 2 to 6 weeks of age [20]. They start to produce eggs by 21 weeks and their egg production rate declines at the age one year and beyond. Aged laying hens were found to have dysbiotic gut microbiota [21]. Furthermore, long-term misuse and abuse of antibiotics has led to gut dysbiosis, induction of antibiotic resistance (AR) and antibiotic resistance genes (ARG) in pathogenic bacteria, and antibiotic residue in chicken eggs and meat, raising public concerns over food safety, antibiotic ineffectiveness, and spreading of zoonotic bacteria [22]. Every year, horizontal and vertical transmission of ARG in bacteria causes 700,000 human deaths; this is expected to rise to 10 million deaths a year by 2050 [23]. Both types of ARG transmission entail multiple molecular mechanisms, involving conjugation and recombination among plasmids, integrons, and chromosomes in bacteria [24]. 

Phytogenics, including plant extracts and compounds, are emerging as an attractive alternative to antibiotics [25]. Over 1340 plants have been claimed to possess anti-bacterial activity [20,26]. Among them, *Bidens pilosa*, an Asteraceae herb, has been reported to modulate gastrointestinal bacteria, increase abundance of seven probiotic genera, decrease abundance of five pathogenic genera, and alleviate protozoal infections in broilers [20]. However, its modulation of gut microbiota in laying hens is not clear. Of note, we and others have identified 301 phytochemicals from this plant [26,27]. Here, we first investigated the beneficial effects of the novel *B. pilosa*-based formulation, EUBIO-BPSG (hereinafter referred to as BP), on mortality, reproductive performance, AR and change in total fecal count, Lactobacillus, E. coli, and S. enterica in 54-week-old laying hens. Next, metagenomic and polymerase chain reaction (PCR) approaches were used to characterize composition of fecal probiotics and pathogens in aged hens. This was followed by investigation of the growth regulation of *L. reuteri, E. coli, and S. enterica* by BP and antagonism among the gut bacteria. *L. reuteri*-producing SCFA and their action on pathogens were identified, and finally, the BP-mediated regulation of conjugation and recombination of ARG in bacteria was studied. 

## 2. Materials and Methods

### 2.1. Chemicals, Reagents, Bacterial Strains, Plasmids, and Media

LA, AA, BA, PA, and 3-HPA (Sigma-Aldrich, Munich, Germany), ^13^C_6_-3NPH·HCl (Cayman Chemical, Ann Arbor, MI, USA) were purchased. The phytogenic formulation, EUBIO-BPSG, composed of *B. pilosa* phytochemicals and glutamate, was manufactured and its quality control was performed by Eubiotics Co. (Taipei, Taiwan). Bacterial strains, plasmids, bacterial media, and PCR primers are listed in Tables S2–S5, respectively.

### 2.2. Animal Study

Fifty-four-week-old Lohmann Brown hens (12,000) were housed and handled in compliance with the Academia Sinica Institutional Animal Care and Use Committee guidelines (protocol No. 18-02-1188). Birds with initial body weight of 1.95 ± 0.06 kg were assigned randomly into 4 groups, 3000 birds per house. Birds were fed daily with a standard diet or the diet containing BP at the indicated doses for 4 weeks (Figure S1A). Cadavers, eggs, and stools were collected at the indicated time points to assess ADM, EPR, and gut microbiota. Alternatively, BP at 250 ppm for the indicated time was used in subsequent experiments unless indicated.

### 2.3. DNA Extraction and NGS Analysis of the Gut Microbiome [28]

To characterize the effects of EUBIO-BPSG on the chicken microbiome, 1–2 g of stool content per chicken (3000) from each group of the CTR and the 4-week fed BP was collected into 2 mL sterile polypropylene tubes at the end of treatment. Then, they were quickly frozen in liquid nitrogen, stored in a freezer at −80 °C overnight for further analysis. Then, all stools were processed at the same time on an ice bucket in the laboratory and the bacterial DNA was extracted from feces of CTR and BP-fed laying hens using PowerFecal Pro DNA Kit (Qiagen, Germany) and kept in a −20 °C freezer until analysis using sequencers on the next day. For 16S rDNA NGS, bacterial DNA was amplified using PCR with 16S rDNA primers and sequenced (NovaSeq sequencing 6000, Illumina, San Diego, CA, USA). The 16S rDNA sequences were trimmed using Chimera Check and analyzed using RDP. Composition of bacterial genera was analyzed using R package (v.3.3.1). For shotgun metagenomic sequencing, bacterial DNA was fragmented, sequenced, and analyzed according to the BioTools protocol (New Taipei, Taiwan). ARG and integrons were scrutinized using the DIAMOND (0.9.22) package against NCBI RefSeq, Comprehensive Antibiotic Resistance Database, and INTEGRAL.

### 2.4. Growth, Counting, and AR Measurement of Fecal Bacteria [29]

Fresh stools were collected from 54-week-old hens fed with standard diet or BP for 2 and 4 weeks. Eight samples were collected for each group at 2 time points, 2 stools per sample. The 16 stools were cultured. Total bacteria, *Lactobacillus*, *E. coli*, and *Salmonella* were grown on plate count agar, MRS, eosin methylene blue, and *Salmonella Shigella* selective plates, respectively, at 37 °C for 3 days under anaerobic conditions. To detect AR in fecal bacteria, the above stools from 58-week-old hens, 21 stools per group, were collected. Each pool had 7 stools and each pool was made of 3 dilutions for bacterial culture on 1/5 TSA agar plates containing cycloheximide (10 µg/mL) plus Car (300 μg/mL), Chl (25 μg/mL), Ery (200 μg/mL), Gen (100 μg/mL), Kan (200 μg/mL), Sul (150 μg/mL), or Tet (60 μg/mL). After 3 days, their colonies were counted [29]. All the NGS data were deposited to the NCBI database under accession numbers, SRR19894949, SRR19894950, SRR21732735, and SRR21732736.

### 2.5. MAC, MIC, IC50, and Disc Diffusion Assays

The MAC and MIC of BP for bacteria were measured in six replicates as published [30]. For MAC tests, *L. reuteri* (5 × 10^5^ CFU/mL) was incubated with BP (2 to 128 µg/mL), Amp (10 µg/mL), and 0.1% methanol (NC) at 37 °C for 10 h. For MIC tests, *E. coli* and *S. enterica* at 5 × 10^4^ CFU/mL were incubated with BP (100 to 6400 µg/mL), Amp (10 µg/mL), and 0.1% methanol at 37 °C for 18 h. Bacterial growth (%) was calculated as ratio of optical density at 600 nm (OD_600_) of the treatment group to that of the NC group multiplied by 100. For disc diffusion assays, *E. coli* and *S. enterica* grown on LB plates were incubated for 14 and 12 h, respectively, with MRS medium (150 µL), LB agar disc, water (5 µL), a paper disc soaked with Chl (1 µg), and supernatant (150 µL) and agar discs of *L. reuteri* grown for 16 h. Alternatively, the same disc diffusion assays were performed for both pathogens, except that each had a mixture of 5 SCFAs, which had the same dosages as those in *L. reuteri* supernatant in MRS broth with or without BP. Likewise, *L. reuteri* grown on MRS plates were incubated with LB medium (150 µL), MRS agar disc, water (5 µL), a paper disc soaked with Chl (1 µg), and agar discs of *E. coli* or *S. enterica* grown for 16 h. The diameter of the inhibition zone was photographed and measured using ImageJ software. The IC_50_ value of each SCFA against both pathogens was determined using OD_600_. 

### 2.6. Measurement of HGT, Conjugation, and Recombination Frequency

A pD042-harboring donor *E. coli* (*pir^+^* β2163) and a recipient *E. coli* (*pir^−^* UB5201), harboring p3938 and p929 plasmids (Tables S2 and S3) were prepared as published [31]. Both donors and recipients were mated in the presence of 0.1% methanol (CTR) and BP (0.5 to 50 μg/mL) on filter membrane at 37 °C for 4 h. The mixture was grown on selection medium for recombinants and recipients (details in Table S4). After 2 days, the bacteria were counted. Eight colonies per group were extracted for their DNA and measured for their positive rate using PCR. The HGT frequency (AU) is presented as a ratio of number of recombinants to number of all recipients × PCR positive rate. Likewise, pD042 was electroporated into a recipient *E. coli* UB5201 carrying p3938 and p929. The bacteria were incubated with 0.1% methanol and BP (0.5, 2.5, 12.5, and 50 μg/mL) at 37 °C for 1 h and then grown on selection medium for recombinants and recipients for 2 days. Eight colonies per group were extracted for DNA and measured for positive rate using PCR. Recombination frequency (AU) is presented as ratio of number of recombinants to number of all recipients × PCR positive rate. For conjugation assays [32], RP4-harboring donors (*E. coli* BM21) and plasmid-free recipients (*E. coli* MG1656) were mated in the presence of 0.1% methanol and BP (0.5 to 50 μg/mL) at 37 °C for 1 h. The bacteria were grown on selection medium for transconjugants and donors (details in Table S4). After 16 h, the bacteria were counted. The conjugation frequency (AU) is presented as the ratio of number of transconjugants to number of donors. Alternatively, in vitro recombination assays were performed using the Gateway cloning system (ThermoFisher, Waltham, MA). Briefly, integrase (Gateway clonase), a *ccdB* suicide gene-carrying pDONR221 vector, and a linearized fragment, either pEXP7-Tet (PC) or DNA carrying an *attB* site linked to Amp-resistance (*amp*^R^) gene were incubated in the absence (NC) and presence of BP (0.5 to 50 μg/mL) at 25 °C for 1 h. After proteinase K digestion, the mixtures were transformed into DH5α *E. coli*. The bacteria were grown on LB plates containing Amp (100 µg/mL) or Tet (20 µg/mL) overnight and counted. 

### 2.7. PI Staining and TEM Analysis [33]

*E. coli* and *S. enterica* (5 × 10^7^ CFU/mL) were treated with Amp (30 μg/mL) and each SCFA at the same dosages as its IC_50_ value for 30 min. Bacteria were stained with PI (20 μg/mL), and analyzed with an LSR II flow cytometer (BD Biosciences, Franklin Lakes, NJ, USA) and FlowJo software. Alternatively, both pathogens were treated with Amp (30 μg/mL) for 30 min and an SCFA mixture at dosages that equaled the composition of the fecal SCFA in BP-fed chickens for 0 to 1 h. The bacteria were divided into two aliquots. One aliquot also underwent PI staining and flow cytometry. The other aliquot was analyzed using TEM (FEI Company, Hillsboro, OR, USA).

### 2.8. Quantification of SCFA in Chicken Feces Using LC-MS/MS

Data from three independent experiments or more are presented as mean ± standard deviation (SD). ANOVA test and log rank were used for statistical analysis of differences between groups, and *p* (*) < 0.05, *p* (**) < 0.01, and *p* (***) < 0.001 are considered statistically significant. Stools from 3-week-old chickens fed with vehicle and 250 ppm BP for 4 weeks were collected. Their aqueous extracts (40 μL) were incubated with ^13^C_6_-3NPH·HCl to conjugate short-chain metabolites in the supernatants as published [34]. The reaction mixtures were analyzed using the Acquity UPLC chromatography coupled to a Xevo TQ-XS mass spectrometer (Waters, Millford, MA, USA) with an ESI source in negative mode.

### 2.9. Statistics

Data from three independent experiments or more are presented as mean ± standard deviation (SD). ANOVA test and log rank were used for statistical analysis of differences between groups, and *p* (*) < 0.05, *p* (**) < 0.01, and *p* (***) < 0.001 are considered statistically significant.

## 3. Results

### 3.1. BP Increases Egg Production and Probiotics and Reduces Mortality, Antibiotic Resistance, and Pathogens in Aged Laying Hens

**Figure 1:**
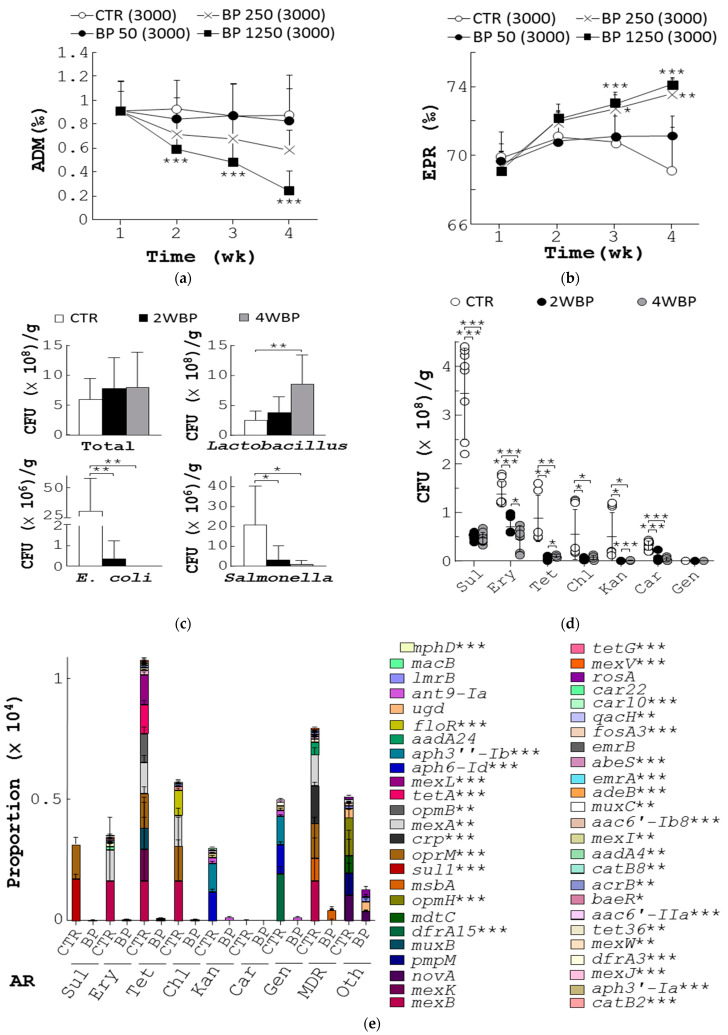
Beneficial effects of BP on animal health, egg production, and gut microbiota in aged laying hens. (**a**,**b**) Four groups of 54-week-old laying hens, 3000 birds a group, were fed with standard diet (CTR) or the diet containing BP at 50 ppm (BP 50), 250 ppm (BP 250), and 1250 ppm (BP 1250) for 4 weeks. Their ADM (**a**) and EPR (**b**) were determined. (**c**) Sixteen stool samples (**a**) from control hens (CTR), aged 58 weeks, and the hens fed with 250 ppm BP for 2 weeks (2 WBP) and 4 weeks (4 WBP), were collected. Total bacteria, *Lactobacillus*, *E. coli*, and *Salmonella* in stools of each group were measured and presented in CFU/gram of stools. (**d**) Bacterial counts of the 21 stools from each group in Figure 1c were grown on agar plates containing the indicated antibiotics. (**e**) The most abundant 50 ARG (right) were classified into 9 common antibiotic categories (left) based on metagenomics analysis of fecal samples from control (CTR) and BP-fed hens (BP), aged 58 weeks. Data from 3 repeats are presented as the mean ± SD. One-way ANOVA test was used for statistical analysis of differences between groups and *p* (*) < 0.05, *p* (**) < 0.01, and *p* (***) < 0.001 are considered statistically significant.

To evaluate the beneficial properties of BP, we first assessed its impact on host health, reproductive performance, bacterial counts, and AR in aged laying hens. Four groups of 54-week-old (12,000) laying hens were fed with a standard feed (control) or feed supplemented with BP at 50 ppm (BP 50), 250 ppm (BP 250), and 1250 ppm (BP 1250) for 4 weeks (Figure S1A). At 58 weeks, control birds had high average daily mortality (ADM) of 0.9‰ (CTR, Figure 1a). However, the birds fed with BP at 50, 250, and 1250 ppm reduced their ADM to 0.8‰, 0.6‰, and 0.2‰, respectively (BP 50, BP 250, and BP 1250, Figure 1a). Accordingly, control hens had an egg production rate (EPR) of 69.1‰ at the age of 58 weeks (CTR, Figure 1b). In contrast, the BP groups had EPR of 71.1‰, 73.6‰, and 74.3‰, respectively (BP 50, BP 250, and BP 1250; Figure 1b). BP at 250 ppm was selected for further experiments due to its cost-effectiveness and efficacy in laying hens. 

Next, we examined the effect of BP on composition and AR of fecal bacteria in laying hens aged 58 weeks. The total count of fecal bacteria seemed to increase over time (Total, Figure 1c). Moreover, BP significantly increased fecal *Lactobacillus* in chickens with 2-week and 4-week treatment (*Lactobacillus*, Figure 1c). Conversely, BP dramatically decreased fecal *E. coli* and *Salmonella* in chickens with 2-week and 4-week treatment (*E. coli* and *Salmonella*, Figure 1c). Of note, fecal bacteria from control birds showed extremely high AR to seven antibiotics, sulfonamide (Sul), tetracycline (Tet), chloramphenicol (Chl) and erythromycin (Ery), carbenicillin (Car), kanamycin (Kan), and gentamicin (Gen) (CTR, Figure 1d). In contrast, fecal bacteria from those fed with BP for 2 and 4 weeks showed a significant dose-dependent reduction in AR (2WBP and 4WBP, Figure 1d). 

Finally, we studied the action of BP on ARG using metagenomic analysis. We first analyzed all ARG in the fecal metagenome of control and BP-fed hens (Figure 1e). All ARG, containing the genes that resist Sul, Ery, Tet, Chl, Kan, Car, Gen, multiple drug class (MDR), and others (Oth), were found in fecal bacteria of both chicken groups (left, Figure 1e). However, a significant reduction of the ARG, as indicated by asterisks, in fecal bacteria by BP was observed (CTR vs. BP, right, Figure 1e). Together, BP made an unexpected beneficial contribution to host health, reproductive performance, amelioration of gut microbiota, and diminution of ARG in aged laying hens. 

### 3.2. BP Differentially Regulates Fecal Bacterial Genera in Aged Laying Hens

**Figure 2:**
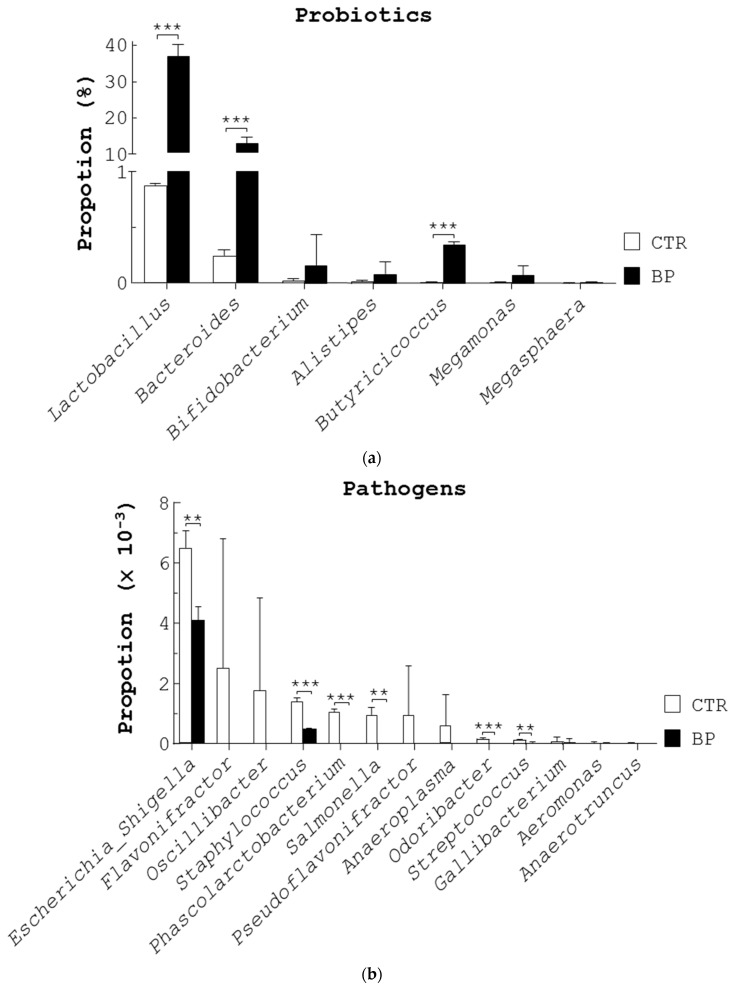
BP increases the 7 probiotic genera and decreases the 13 pathogenic genera. The stools of 54-week-old laying hens (Figure 1c) that were given a standard diet (CTR) and the diet containing 250 ppm BP for 4 weeks were collected to obtain fecal bacteria DNA, followed by bacterial 16S rDNA NGS analysis. The proportion of probiotics (**a**) and pathogens (**b**) of the fecal microbiota were analyzed at the genus level. Data from 3 repeats are presented as the mean ± SD. One-way ANOVA test was used for statistical analysis of differences between groups and *p* (**) < 0.01 and *p* (***) < 0.001 are considered statistically significant.

Next, we compared the composition of fecal bacteria in control and BP-fed birds (Figure 1) using 16S rDNA next generation sequencing (NGS) analysis. First, we analyzed the operational taxonomic units (OTUs). The number of sequences, OTUs, and diversity indices in the feces of both bird groups are summarized in Table S1. Rarefaction curves showed that the number of sequences from two bird groups were enough to uncover the major OTUs (Figure S1B). The fecal microbiota of control hens (CTR), aged 58 weeks, had higher diversity than BP-fed hens (BP) as evidenced by Shannon and Chao1 diversity indices (Table S1). Detailed changes at the order, family, and genus levels under BP treatment are shown in Table S1 and Figure S1C. Seven beneficial bacterial genera, *Lactobacillus* (75.1%)*, Bacteroides* (20.9%), *Bifidobacterium* (1.7%), *Alistipes* (1.6%), *Butyricicoccus* (0.4%), *Megamonas* (0.3%), and *Megasphaera* (0.1%), were most abundant in the control stools (CTR, Figure 2a). BP increased the above seven genera though only *Lactobacillus*, *Bacteroides*, and *Butyricicoccus* were statistically significant (BP, Figure 2a). In contrast, 13 harmful bacterial genera, *Escherichia/Shigella* (40.5%), *Flavonifractor* (15.6%), *Oscillibacter* (11%), *Staphylococcus* (8.5%), *Phascolarctobacterium* (6.5%), *Salmonella* (5.9%), *Pseudoflavonifractor* (5.9%), *Anaeroplasma* (3.7%), *Odoribacter* (0.9%), *Streptococcus* (0.7%), *Gallibacterium* (0.5%), *Aeromonas* (0.2%), and *Anaerotruncus* (0.1%) were most abundant in the control stools (CTR, Figure 2b). Notably, BP reduced the above 13 genera though only *Escherichia/Shigella*, *Staphylococcus*, *Phascolarctobacterium*, *Salmonella*, *Odoribacter*, and *Streptococcus* were statistically significant (BP, Figure 2b).

Next, shot-gun metagenomics NGS was applied to analyze the composition of bacterial species of interest. Ten *Lactobacillus* species and four *Bifidobacterium* species were identified from the chicken stools. They were upregulated by BP (Figure S2A). Semi-quantitative PCR was used and confirmed the upregulation of *L. reuteri* and *L. oris* (Figure S2B,C). Conversely, *E. coli* and *S. enterica* were significantly downregulated by BP in both the metagenomics and PCR data (Figure S2D,F). *L. reuteri*, *E. coli*, and *S. enterica* in the stools were isolated (Figure S3A) and confirmed using mass spectrometry (Figure S3B). Overall, BP increased beneficial probiotics but decreased pathogenic bacteria in chicken stools.

### 3.3. Promotion of Probiotics and Inhibition of Pathogenic Bacteria by BP In Vitro

**Figure 3:**
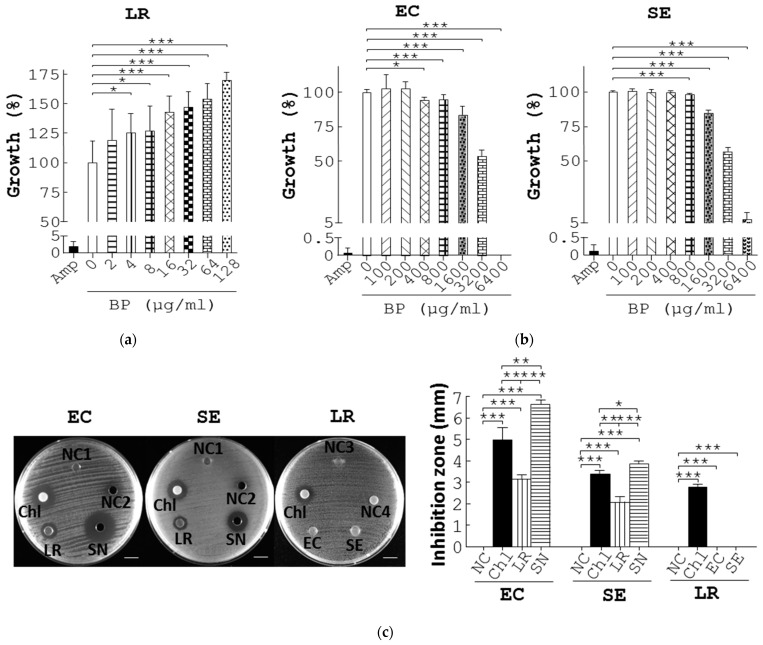

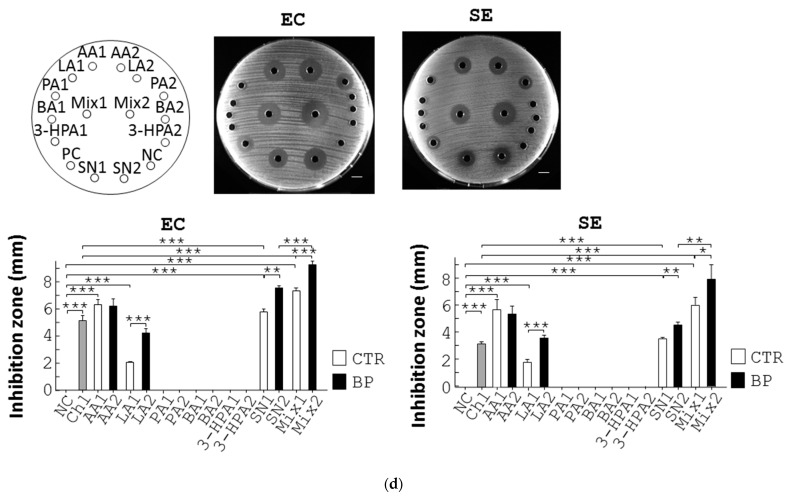
BP suppresses pathogenic growth via upregulation of *L. reuteri* growth and its antimicrobial metabolites. (**a**) *L. reuteri* (LR) was cultured in MRS medium containing Amp, 0.1% methanol (0), and BP at the indicated dosages at 37 °C under anaerobic conditions for 10 h. The growth rate (%) of bacteria was obtained from the ratio of the OD_600_ of the treatment group to that of the control group multiplied by 100%. (**b**) The same procedure as (**a**) was performed except that *E. coli* (EC) and *S. enterica* (SE) were grown in LB broth containing Amp, 0.1% methanol (0), and BP at the indicated dosages. (**c**) Antagonisms between *L. reuteri* and pathogens. *E. coli* (EC, 1st column, left) and *S. enterica* (SE, 2nd column, left) were spread on LB plates and incubated with a MRS agar disc (NC1), 150 µL MRS medium (NC2), a paper disc containing 1 µg Chl, *L. reuteri* grown on an agar disc (LR), and 150 µL supernatant of *L. reuteri* (SN). *L. reuteri* (LR, 3rd column, left) was spread on MRS plates and incubated with a LB agar disc (NC3), 5 µL water (NC4), a paper disc containing Chl (1 µg), and *E. coli* (EC) and *S. enterica* (SE) grown on agar discs. Scale bar = 1 cm. The diameter (mm) of their inhibition zones was measured and replotted into histograms (right). (**d**) The labelling of the treatment groups in the plate is indicated (top left). *E. coli* (EC, top middle), and *S. enterica* (SE, top right) were spread on LB plates and incubated with 150 µL MRS broth (NC), Chl (1 µg), AA (462.3 µg, AA1), LA (222.97 µg, LA1), PA (0.29 µg, PA1), BA (0.56 µg, BA1), 3-HPA (1.80 µg, 3-HPA1), a mixture of AA1, LA1, PA1, BA1, and 3-HPA1 (mix1), the supernatant of *L. reuteri* culture (SN1), AA (440.13 µg, AA2), LA (715.33 µg, LA2), PA (0.23 µg, PA2), BA (0.7 µg, BA2), 3-HPA (5.3 µg, 3-HPA2), a mixture of AA2, LA2, PA2, BA2, 3-HPA2 (mix2), and the supernatant of *L. reuteri* cultured with BP (SN2) for 16 h. Scale bar = 1 cm. The diameter (mm) of their inhibition zones was measured and replotted into histograms (bottom). Data from 3 repeats are presented as the mean ± SD. One-way ANOVA tests were used for statistical analysis of differences between groups and *p* (*) < 0.05, *p* (**) < 0.01, and *p* (***) < 0.001 are considered statistically significant.

To dissect mechanisms whereby BP differentially regulated growth of probiotics and pathogens, we first grew *L. reuteri* in Rogosa and Sharpe (MRS) medium supplemented with BP. As expected, Amp (10 µg/mL), a positive control, reduced *L. reuteri* growth by 95% compared to the vehicle (Amp vs. 0 µg/mL BP, Figure 3a). In contrast, BP dose-dependently promoted *L. reuteri* growth (4 to 128 µg/mL BP, Figure 3a) and its minimum activating concentration (MAC) was 4 µg/mL. In parallel, we grew *E. coli* and *S. enterica*, in Luria–Bertani (LB) medium containing BP. As expected, Amp (10 µg/mL) reduced *E. coli* growth by 99% compared to the vehicle (Amp vs. 0 µg/mL BP, EC, Figure 3b). BP also dose-dependently inhibited *E. coli* growth (100 to 6400 µg/mL BP, EC, Figure 3b) Likewise, BP inhibited *S. enterica* growth (SE, Figure 3b). Furthermore, BP had a minimum inhibitory concentration (MIC) value of 400 and 800 µg/mL for *E. coli* and *S. enterica*, respectively (Figure 3b). 

Collectively, the MAC and MIC data demonstrated an opposite regulation of growth of probiotics and pathogens by BP in vitro. A 100-fold difference in MAC and MIC of BP suggested that BP directly promoted probiotic growth and, therefore, led to a reduction in pathogens.

### 3.4. BP Inhibits Pathogenic Bacteria through Upregulation of SCFA in L. reuteri

To decode the mechanism by which BP inhibited pathogens through the action of probiotics, we first tested the antagonism between *L. reuteri* and *E. coli* or *S. enterica*. Disc diffusion assays showed no inhibition on *E. coli* growth using discs containing MRS agar and MRS medium (NC1 and NC2, EC, Figure 3c). However, the positive control, chloramphenicol (Chl), exhibited an obvious inhibition zone on *E. coli* (Chl, EC, Figure 3c). Likewise, an agar disc containing *L. reuteri* colonies suppressed the growth of *E. coli* (LR, EC, Figure 3c). Further, the *L. reuteri* supernatant inhibited growth of *E. coli* to a greater extent than its colonies (SN vs. LR, EC, Figure 3c). It was the case for regulation of *S. enterica* by *L. reuteri* (SE, Figure 3c).

Next, we examined whether both *E. coli* and *S. enterica* could inhibit *L. reuteri*. Although Ch1 and both negative controls worked as expected, *E. coli* and *S. enterica* failed to suppress growth of *L. reuteri* (LR, Figure 3c). The data demonstrated direct antagonism of *E. coli* and *S. enterica* by *L. reuteri* supernatants and colonies but not vice versa. Thus, this interbacterial antagonism was mediated in a cell contact-independent manner.

The cell contact-independent antagonism between *L. reuteri* and pathogens prompted us to identify antimicrobial components produced by *L. reuteri*. Ultra-performance liquid chromatograph mass spectrometry (UPLC/MS) was used to seek for its anti-bacterial metabolites. We confirmed and quantified 5 SCFAs, acetic acid (AA), lactic acid (LA), propionic acid (PA), butyric acid (BA), and 3-hydroxypropionaldehyde (3-HPA) in *L. reuteri* supernatant in comparison with their standards (Figure S4A). Furthermore, UPLC/MS revealed that BP significantly upregulated the level of the 5 SCFAs over time (Figure S4A). We then tested the antimicrobial effects of the 5 SCFAs on *E. coli* and *S. enterica* using disc diffusion assays. No inhibition of *E. coli* growth was observed in a disc filled with distilled water but the opposite was for Chl (NC vs. Chl, EC, Figure 3d), while the supernatant of *L. reuteri* showed inhibition of *E. coli* growth (SN1, EC, Figure 3d) to a lesser degree than that of *L. reuteri* with BP treatment (SN2, EC, Figure 3d). We also evaluated the antimicrobial potency of the 5 SCFAs, individually or in combination, at the dosage that equaled their quantity in the supernatant of *L. reuteri*. PA, BA, and 3-HPA at the indicated dosages showed no inhibition of *E. coli* growth (PA1, BA1, and 3-HPA1, EC, Figure 3d), whilst LA, AA, and a mixture of the 5 SCFAs showed significant inhibition of *E. coli* growth (LA1, AA1, and Mix1, EC, Figure 3d). In addition, we tested the antimicrobial potency of the 5 SCFAs, individually or combinationally at the dosage that equaled their quantity in the *L. reuteri* supernatant with 16-h BP treatment. Likewise, PA, BA, and 3-HPA at the indicated dosage showed no inhibition against *E. coli* growth (PA2, BA2, and 3-HPA2, EC, Figure 3d), whilst LA, AA, and a mixture of the 5 SCFAs showed significant inhibition of *E. coli* growth (LA2, AA2, and Mix2, EC, Figure 3d). Similarly, LA, AA, and a mixture of the 5 SCFAs (LA2, AA2, and Mix2, Figure 3d), but not PA, BA, and 3-HPA, showed significant inhibition of *S. enterica* growth (PA2, BA2, and 3-HPA2, SE, Figure 3d). Obviously, LA (LA2), a mixture of the 5 SCFAs (Mix2), and supernatant of BP-treated *L. reuteri* (SN2) corresponding to their amount in BP treatment cases, had superior inhibition of *E. coli* and *S. enterica* (LA1, Mix1, and SN1), corresponding to their amount in control cases (EC and SE, Figure 3d).

Overall, BP antagonized growth of *E. coli* and *S. enterica* via upmodulation of SCFA production from *L. reuteri*. 

### 3.5. SCFA Suppress Growth of Pathogenic Bacteria via Membrane Destruction

**Figure 4:**
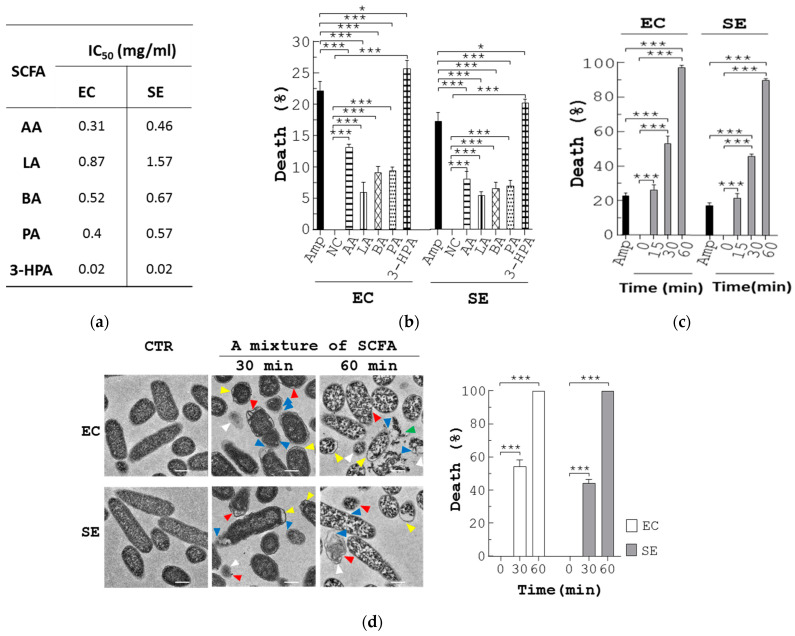

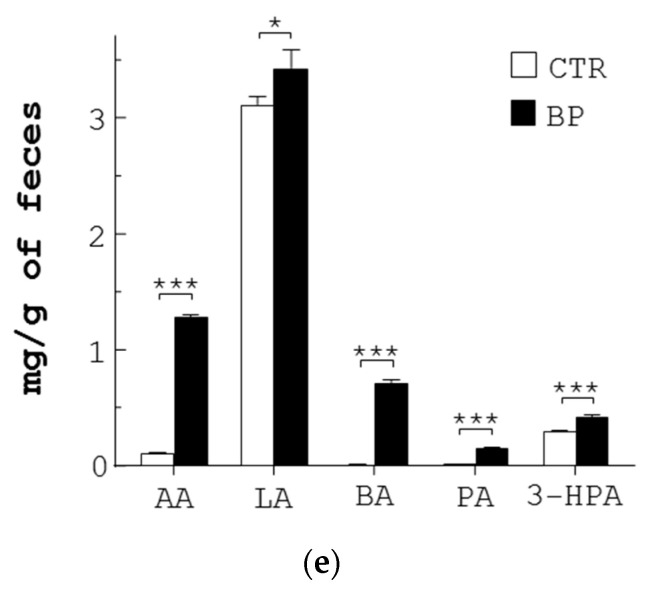
Anti-pathogenic mechanism of SCFA produced from *L. reuteri*. (**a**) The IC_50_ of each SCFA for *E. coli* (EC) and *S. enterica* (SE) was determined. (**b**) Each of the pathogens (5 × 10^7^ CFU/mL) were grown in LB broth containing vehicle (NC), Amp (30 µg/mL), and each SCFA at its IC_50_ dose for 30 min, followed by PI staining. The bacteria were analyzed with flow cytometry to determine the percentage (%) of dead cells. (**c**,**d**) *E. coli* (EC) and *S. enterica* (SE) were treated with Amp and a mixture of the 5 SCFAs at the same doses as those in the feces of BP-fed chickens, for the indicated times. One aliquot of the bacteria was stained with PI and analyzed with flow cytometry to determine the percentage (%) of dead cells (**c**). The other aliquot of *E. coli* (EC) and *S. enterica* (SE) was analyzed using TEM. Their representative images are shown (left, **d**). Arrowheads in yellow, red, white, and blue indicate separation of the cytoplasmic and outer membranes, distorted outer membrane, empty cells, and membrane discontinuity, respectively. Their death (%) was quantified and replotted into histograms (right, **d**). Scale bar = 0.5 µm. (**e**) Three-week-old chickens (*n* = 3) were fed with standard diet (CTR) and the diet containing 250 ppm BP for 4 weeks. Fecal extracts were subjected to LC-MS/MS analysis, followed by quantification of 5 SCFAs (µg/g of feces). Data from 3 repeats are presented as the mean ± SD. One-way ANOVA tests were used for statistical analysis of differences between groups and *p* (*) < 0.05 and *p* (***) < 0.001 are considered statistically significant.

To better understand the antimicrobial mode of action of these SCFA, we assessed the bactericidal activities of individual SCFA towards *E. coli* or *S. enterica*. The half maximal inhibitory concentration (IC_50_) values of the SCFA against *E. coli* in ascending order were: 3-HPA (0.02 mg/mL) < AA (0.31 mg/mL) < PA (0.4 mg/mL) < BA (0.52 mg/mL) < LA (0.87 mg/mL) (EC, Figure 4a). The IC_50_ values of the SCFA against *S. enterica* in ascending order were: 3-HPA (0.02 mg/mL) < AA (0.46 mg/mL) < PA (0.57 mg/mL) < BA (0.67 mg/mL) < LA (1.57 mg/mL) (SE, Figure 4a). Next, we investigated cell death of *E. coli* and *S. enterica* using a flow cytometer. No cell death of *E. coli* in MRS medium was shown by propidium iodide (PI) staining (NC, EC, Figure 4b). However, Amp induced 22% death in *E. coli* (Amp, EC, Figure 4b). Consistently, each SCFA caused a different degree of death in *E. coli* cells (AA, LA, 3-HPA, PA, and BA, EC, Figure 4b). Meanwhile, Amp induced 17% death in *S. enterica* (Amp, SE, Figure 4b). Each SCFA caused a different degree of death in *S. enterica* (AA, LA, 3-HPA, PA, and BA, SE, Figure 4b). In short, the percentage of cell death in the two pathogens caused by each SCFA was in good agreement with their IC_50_ values. Next, we explored the antimicrobial action of a mixture of the 5 SCFAs at the dosage that equaled their quantity in the feces of BP-fed chickens. Similar to Amp, this mixture increased cell death in both *E. coli* and *S. enterica* cells over time (EC and SE, Figure 4c). Transmission electron microscopy (TEM) showed that, in the absence of the mixture of 5 SCFAs, the control bacteria had intact membranes and regular cytoplasm (CTR, EC, and SE, Figure 4d). In marked contrast, 30 min treatment with the mixture induced damaged/dead cells by 54% and 44% in *E. coli* and *S. enterica*, respectively (SCFA/30 min, EC and SE, Figure 4d). Likewise, this percentage increased over time (SCFA/60 min, EC, and SE, Figure 4d). 

We also confirmed the presence of the 5 SCFAs in stools of control and BP-fed chickens. The UPLC/MS data showed that the amount of 5 SCFAs per gram of control chicken stools in descending order was: LA (3.11 mg) > 3-HPA (0.3 mg) > AA (0.11 mg) > BA ≈ PA (0.01 mg) (CTR, Figure 4e). In contrast, the amount of 5 SCFAs per gram of BP-fed chicken stools in descending order was: LA (3.43 mg) > AA (1.29 mg) > BA (0.72 mg) > 3-HPA (0.43 mg) > PA (0.16 mg) (BP, Figure 4e). Over 4 weeks, BP upregulated the level of AA, PA, and BA to a greater extent than LA and 3-HPA in chicken guts. Overall, BP significantly escalated the level of fecal SCFA in chickens. 

### 3.6. BP Decreases ARG in Fecal Bacteria and Conjugation and Recombination in E. coli

**Figure 5:**
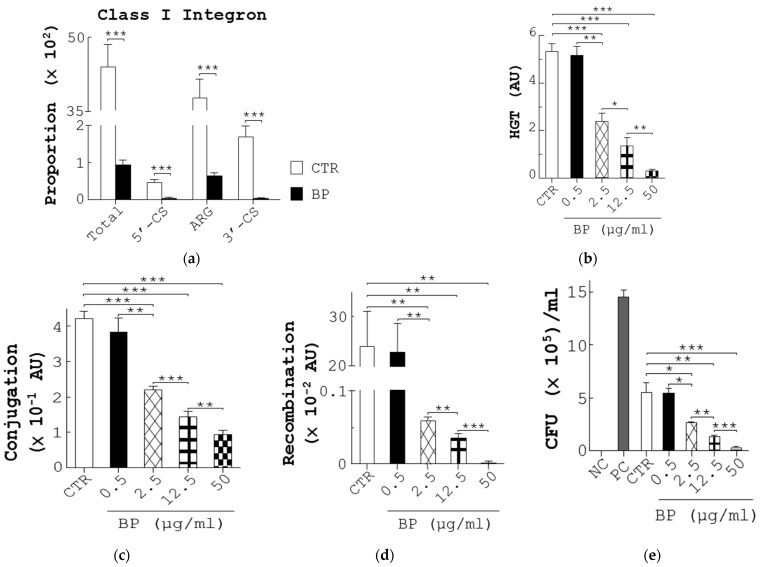

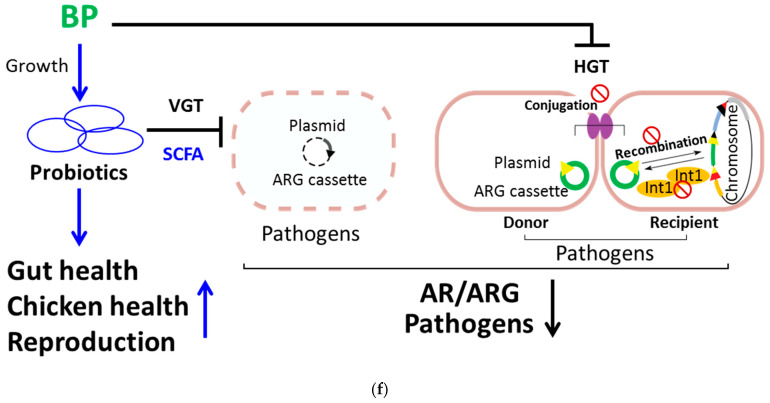
BP reduces the proportion of ARG in fecal bacteria as well as the frequency of bacterial conjugation and recombination. (**a**) The abundance of the class 1 integron (Total), comprising the 5′-CS, ARG, and 3′-CS, in fecal microbiota from control (CTR) and BP-fed hens (Figure 1e) was determined using metagenomic NGS analysis. (**b**) The frequency (AU) of horizontal gene transmission (HGT) was evaluated in each group by mating pD042-harboring donors (β2163) with p3938 and p929-harboring recipients (UB5201) in the presence of 0.1% methanol (CTR) or BP at the indicated dosages. (**c**) The conjugation frequency (AU) was determined by mating RP4-harboring donors (BM21) with recipients (MG1656) in the presence of 0.1% methanol (CTR) or BP at the indicated dosages. (**d**) The recombination frequency (AU) was determined by transforming pD042 into recipients (UB5201) harboring p3938 and p929, followed by incubation in the presence of 0.1% methanol (CTR) or BP at the indicated dosages. (**e**) The inhibition of BP for integrase was analyzed using the Gateway system. One reaction in which pDONR221 and pEXP7-Tet were incubated in the absence (NC) and presence (PC) of integrase (Gateway clonase) were transformed into DH5α. The other reactions in which pDONR221, *attB*-flanked *amp*^R^ DNA, and integrase were incubated with 0.1% methanol (CTR) and BP at the indicated dosages were transformed into DH5α. After overnight growth, the colonies were calculated. Data from 3 repeats are presented as the mean ± SD. One-way ANOVA tests were used for statistical analysis of differences between groups and *p* (*) < 0.05, *p* (**) < 0.01, and *p* (***) < 0.001 are considered statistically significant. (**f**) A scheme illustrating the mechanism of BP for regulation of gut microbiota and beneficial outcomes in laying hens. On one hand, BP promotes probiotic growth and production of its antimicrobial metabolites (e.g., SCFA) and, in turn, promotes gut health, host health and reproductive performance. In addition, SCFA can kill pathogens and this killing reduces VGT of ARG in pathogens. On the other hand, BP also suppresses interbacterial HGT by reduction of conjugation and recombination and enzymatic inhibition of integrase in pathogens.

To explore the mechanism by which BP decreased AR in fecal bacteria, we first analyzed the composition of genes corresponding to class 1 integrons, implicated in ARG transfer in bacteria, in the fecal microbiome of chickens (Figure S5A). Unexpectedly, a high proportion of the total sequencing reads corresponding to the class 1 integrons, composed of 5′-conserved segment (5′-CS), ARG, and 3′-conserved segment (3′-CS), were found in the control fecal microbiome (Total, 5′-CS, ARG, and 3′-CS, CTR, Figure 5a). Conversely, BP profoundly reduced the proportion of total reads and the elements of the class 1 integrons, suggesting a reduction of ARG by BP (BP, Figure 5a). Next, to study the action of BP on HGT of ARG, we first adopted an interbacterial conjugation and recombination system composed of a donor *E. coli* strain (β2163), harboring pD042 plasmid, and a recipient *E. coli* strain (UB5201), harboring p3938 and p929 plasmids, as published [31]. A co-incubation of donors and recipients in this system generated an HGT frequency of 5.4 arbitrary units (AU) (CTR, Figure 5b). In contrast, BP dose-dependently reduced this frequency (BP, Figure 5b). To further pinpoint the action of BP on conjugation and recombination steps, three assay platforms were applied. The first was a conjugation platform composed of a donor *E. coli* strain (BM21), harboring RP4 plasmid, and a plasmid-free recipient *E. coli* strain (MG1656 (Δ*dapA*(::*frt*) *recA*^−^(Tn*10*))) as published [32]. A co-incubation of donors and recipients in the assays generated a conjugation frequency of 4.2 × 10^−1^ AU (CTR, Figure 5c). In contrast, BP dose-dependently reduced this frequency (BP, Figure 5c). The second platform was a recombination platform composed of a donor plasmid, pD042, and a recipient *E. coli* strain (UB5201), harboring p3938 and p929 plasmids, as published [31]. After pD042 was transformed into recipients, the recombination frequency of the recipients in vehicle groups was 23.9 × 10^−2^ AU (CTR, Figure 5d). In contrast, BP dose-dependently reduced this frequency (BP, Figure 5d). The third platform was to use the Gateway recombination system to assess the action of BP on integrase activity. As expected, no recombination was observed in the mixture of pEXP7-Tet and pDONR221 in the absence of integrase (NC, Figure 5e). However, there was a recombination frequency of 14.6 × 10^5^ CFU/mL in the mixture of pEXP7-Tet, a positive control, pDONR221, and integrase (PC, Figure 5e). Similarly, there was a recombination frequency of 5.6 × 10^5^ CFU/mL in the mixture of the DNA comprising *attB* and *amp^R^* (CTR), pDONR221, and integrase (CTR, Figure 5e). In contrast, BP dose-dependently reduced this recombination frequency (BP, Figure 5e), suggesting the inhibition of integrase activity by BP. Taken together, BP reduced ARG in bacteria via downregulation of HGT involving inhibition of conjugation, recombination, and integrase.

In conclusion, a dysregulation of gut microbiota, augmented pathogens and diminished probiotics, and affected AR, host health, and reproductive performance in aged laying hens. Nevertheless, BP could ameliorate the dysregulation of gut microbiota, AR, host health, and reproduction in birds (Figure 5f). Mechanistically speaking, BP promoted growth of probiotics (e.g., *L. reuteri*) and, in turn, increased gut health, host health, and reproduction of laying hens. Meanwhile, BP upregulated antimicrobial metabolites (e.g., SCFA) of probiotics, which directly killed pathogens (e.g., *E. coli* and *S. enterica*), and subsequently, reduced VGT in pathogens. Besides, BP downregulated HGT in pathogens through suppression of bacterial conjugation and recombination and enzymatic inhibition of integrases (Figure 5f).

## 4. Discussion

Medicinal plants and their compounds improve health, growth, and/or reproduction in laying hens [35,36], broilers [37,38], and aged chickens [39]. However, little is known about the mechanisms by which plants/compounds modulate gut microbiota and enhance health and reproduction in animals [36,40,41]. This work demonstrated that BP ameliorated host health, reproductive performance, and AR in aged laying hens by the Yin and Yang balance of gut microbiota, i.e., increasing probiotics and decreasing pathogens. Moreover, BP reduced AR due to inhibition of VGT and HGT of ARG in gut microbiota.

BP improves growth performance and coccidiosis in 1-month-old broilers through manipulation of gut microbiota, including seven increased probiotic genera and five decreased pathogenic genera [20]. The health and reproduction of aged hens declines over time due to dysregulated gut bacteria. This study illustrated that BP dose-dependently improved ADM, EPR, and AR (Figure 1), associated with promotion of seven probiotic genera and diminution of 13 pathogenic genera in the intestines of aged hens (Figure 2). Surprisingly, BP elevated growth of beneficial probiotics and production of antimicrobial metabolites, e.g., SCFA, and, as a result, harmful bacteria were compromised by the SCFA (Figure 3 and Figure 4). Consistently, feed addition of seven organic acids (LA, AA, PA, BA, etc.) to old hens improved EPR and prolonged their laying period [12]. The 5 SCFAs were also detectable in chicken feces (Figure 4e). Of note, limitations of this experiment might affect final concentrations of the SCFA including sample size was only three chickens per group and the procedure of fecal extraction might lead to the loss of these volatile compounds prior to LC-MS/MS analysis. Hence, it is not hard to extrapolate that BP ameliorates gut health, host health, and reproductive performance in aged hens via SCFA-mediated antagonism between probiotics and pathogens (Figure 5f). Moreover, BP also lowered AR in chickens by impairing bacterial VGT and HGT. This BP-implicated mechanism is further supported by publications stating that increased *Lactobacillus* improved chicken health and inhibited growth of pathogens [42,43]. Conversely, the rise in abundance of pathogenic *Escherichia* and *Salmonella* in chicken guts was associated with host pathology [44] and ARG acquisition and transfer [45]. Our research may thus develop novel therapeutics to treat gut pathogens and reduce AR in animals and humans.

Manipulating gut microbiota is a promising strategy to prevent and treat diseases. However, several technical barriers limited our study. One challenging technical barrier was that the in vivo interaction among BP, gut bacteria, and the gut is too complicated to be dissected as a whole. Another barrier was that most intestinal bacteria cannot culture outside the gut. Therefore, a limited number of gut bacteria were used to prove our concept. BP at 4 μg/mL significantly increased growth of *L. reuteri* (Figure 3a). Conversely, BP at 400 and 800 μg/mL, significantly inhibited growth of *E. coli* and *S. enterica,* respectively (Figure 3b). These data suggest that BP reduced both pathogens via indirect antagonism between *L. reuteri* and either of *E. coli* and *S. enterica* (Figure 3c,d). Of note, the dosage of BP used for *L. reuteri* but not *E. coli* and *S. enterica* was easily achievable in the chicken gut (Figure 4e) because the content of 250 ppm BP, an in vivo effective dose for ADM and EPR, roughly equaled 250 μg/g of feed (~250 μg/mL). Furthermore, the data on the BP-mediated regulation of SCFA production in *L. reuteri* (Figure S4) and SCFA mechanism towards pathogens (Figure 4) supported this indirect antagonistic interaction. Of note, the BP-mediated regulation of gut microbiota in aged hens are similar to that of gut microbiota in 1-month-old broilers in terms of bacterial genera [20]. This modulation of gut microbiota by BP is in line with one publication describing plant extracts promoted the population of non-pathogens and concurrently possessed antibacterial activity [46]. However, we cannot rule out the possibility that BP decreased pathogens directly via its antimicrobial phytochemicals since 18 of the 301 phytochemicals identified from BP were reported [47]. Given the complexity of phytochemicals in BP [26], active compounds that upmodulate probiotics, impair HGT and/or VGT, and downmodulate pathogens may not be the same and need to be identified from BP. As expected, each SCFA in chicken guts was effective enough against *E. coli* and *S. enterica* since each SCFA in the chicken stools was at least 30 times higher than their IC_50_ values in *E. coli* and *S. enterica*.

VGT and HGT are two common mechanisms for acquisition of ARG in bacteria [48]. The former is involved in bacterial ARG transmission from parents to off-spring. The latter is implicated in ARG transmission from one bacterium to another, including conjugation and recombination [49]. The application of specific conjugation inhibitors [50], antibiotic degradases (e.g., ribaxamase) [51], or antibiotic absorbents (e.g., DAV132) [52] reduces ARG dissemination in pathogens. However, they are not clinically available. We showed that BP significantly reduced AR (Figure 1e) and ARG of the class 1 integrons in aged chickens (Figure 5a). On one hand, we found that BP increased the production of 5 SCFAs in *L. reuteri* (Figure S4) and the SCFA killed pathogenic bacteria via cell membrane damage (Figure 4). This bacterial killing could stop ARG transmission via the VGT mechanism in pathogens. On the other hand, BP suppressed ARG transmission between *E. coli* via inhibition of the HGT mechanism, including bacterial conjugation and recombination as well as enzymatic inhibition of integrases (Figure 5). Obviously, inhibition of VGT and HGT by BP was not ascribed to the bactericidal action of BP since its dosages had the same viability in bacteria as the vehicle control (Figure S5B–E). The details about active compounds of BP and their molecular targets await further elucidation. 

## 5. Conclusions

The data demonstrate an imbalance of gut microbiota, e.g., an increased ratio of pathogens to probiotics, increased AR, lowered host health, and reproductive performance in aged laying hens. In contrast, BP could improve the dysregulation of gut microbiota, AR, health, and reproduction in aged birds. This improvement involved a BP-mediated rectification of dysbiosis to eubiosis in avian gastrointestinal microbiota. Accordingly, BP upregulated antimicrobial metabolites (e.g., SCFA) of probiotics and, in turn, antagonized growth of pathogens, leading to a reduction of VGT and HGT. This study also reveals the superiority of using phytogenics as the One Health approach replace antibiotics and their bad outcomes. In this concept, we propose to investigate the difference of AR between old chickens and young chicks and chicken farmers to non-farmers to understand more about the transmission of AR among animals, humans, and the environment. Moreover, targeting the coexisting microorganisms is also important that the use of phytogenics should be observed within the interaction of polymicrobial community as a potential approach against diseases.

## Data Availability

The raw data supporting the conclusions of this manuscript are included in this manuscript and Supplementary Information and NCBI databases.

## Supplementary Materials

The following supporting information can be downloaded at: https://www.mdpi.com/article/10.3390/bioengineering10030346/s1.
