# Supplementary material for: A Novel Phytogenic Formulation, EUBIO-BPSG, as a Promising One Health Approach to Replace Antibiotics and Promote Reproduction Performance in Laying Hens
Source: Bioengineering (Basel). 2023 Mar 10;10(3):346. doi: 10.3390/bioengineering10030346 (PMC10045918; doi:10.3390/bioengineering10030346)
Supplement: Supplementary file 1 [file bioengineering-10-00346-s001.zip › bioengineering-2248046-supplementary.pdf]

A novel phytogenic formulation, EUBIO-BPSG, as a promising One Health approach to reducing antibiotic use and its negative impact and promote reproduction performance in laying hens

Hieu Tran Nguyen Minh[1,,2#], Tien-Fen Kuo[1#],Wen-Yu Lin[3], Tzu-Chia Peng[1], Greta Yang[1], Chih-Yu Lin[1], Ting-Hsiang Chang[1], Yu-Liang Yang[1], Cheng-Hsun Ho[1], Bor-Rung Ou[3], Chu-Wen Yang[4*], Wen-Chin Yang[1,2,5,6,7*]and Yu-Chuan Liang[1,3]*

MATERIALS AND METHODS

DNA extraction, NGS analysis, and PCR confirmation of gut microbiota.

The DNA extraction and NGS analysis of 16S rDNA and gut microbiome from laying hens fed with standard diet or the diet supplemented with BP (250 ppm) were performed as described in the Materials and Methods section of the manuscript. Species-specific primers located within 16S rRNA, *tuf*, *yaiO*, and *hisJ* genes were used in PCR to characterize *L. reuteri*, *L. oris*, *E. coli*, and *S. enterica*, respectively.

Isolation, selection, and mass spectrometry-based identification of fecal bacteria.

Fresh stools were collected from aged hens fed with a standard diet and pooled. After a serial dilution, the gut bacteria were grown on pre-reduced agar plates. One set of media was incubated at 37°C for 3 days in an anaerobic chamber. The isolation and selection of *Lactobacillus*, *E. coli* and *Salmonella* were conducted on the selective plates as published[51] under anaerobic conditions at 37°C for 3 days. The procedure of bacterial isolation and identification was delineated in Fig. S3. Briefly, 16 to 37 isolates were subjected to matrix-assisted laser desorption ionization-time of flight (MALDI-TOF) analysis (Bruker, Billerica, MA). The spectral profiles were used to compare with microbial protein profile databases using an in-house database containing over 4,000 microbial strain protein profiles.

Identification of SCFA using the LC-MS/MS.

A chemical derivatization method was used to identify and quantify short-chain metabolites as published[52]. Briefly, the supernatants (40 µl) were collected from *L. reuteri* in co-culture with BP (4 µg/ml) or vehicle (0.1% MeOH) in MRS medium for 5, 8, and 16 h. The samples were incubated with $^{13}C_6$-3NPH·HCl to conjugate short-chain metabolites present in the supernatants. The reaction mixtures were analyzed using Acquity UPLC chromatography coupled to a Xevo TQ-XS mass spectrometer (Waters, Millford, MA) with an ESI source in a negative mode (m/z 50 to m/z 250). The identity of each SCFA was based on their MS1 profiles and the quantification of each SCFA was based on their MS2 profiles.

Measurement of bacterial viability.

Four *E. coli* strains (5×10^4) were tested for their viability in LB broth in the presence of an antibiotic, either Amp (100 µg/ml) or Tet (25 µg/ml), and BP at 0 (NC), 0.5, 2.5, 12.5, and 50 µg/ml at 37°C for 18 h. The bacterial growth rate (%) was calculated by the ratio of the optical density at 600 nm (OD_{600}) of the treatment to that of the NC multiplied by 100%.

SUPPLEMENTARY TABLES

Table S1. The number of sequences, OTUs, taxonomy (phylum, class, order, family, and genus), richness (Chao1), and diversity (Shannon index) in 16S rDNA data obtained from the control (CTR) and BP-fed (BP) fecal samples.

Sample DNA	Sequence No.	OTUs	Phylum	Class	Order	Family	Genus	Chao1	Shannon index
CTR	46490	418	11	18	48	94	143	442	6.07
BP	46490	159	6	13	26	46	83	186.08	4.27

The data are the same as Fig. 2.

Table S2. Bacterial strains used in this study.

Strains	Characteristic	Source
Bacterial strains used for functional and mechanistic studies		
L. reuteri	F275 [DSM 20016]	ATCC 23272
E. coli	FDA strain Seattle 1946 [DSM 1103]	ATCC 25922
S. enterica	NCTC 4444	ATCC 4931
E. coli stains used for conjugation and recombination assays		
β2163	*pir*$^{+}$ donor harboring pD042	[30]
UB5201	*pir*$^{-}$ recipient harboring p929 and p3938	[30]
E. coli stains used for recombination assays		
UB5201	*pir*$^{-}$ recipient harboring p929 and p3938	[30]
E. coli stains used for conjugation assays		
BM21	F^{-} donor harboring RP4	[31]
MG1656	Plasmid-free F^{+} recipient (Δ*dapA*(::*frt*) *recA*$^{-}$	[31]

	(Tn*10*))	
Strains used *in vitro* recombination assay by Gateway reaction		
DH5α	*supE44* Δ*lacU169* (Φ80*lacZ'*Δ*M15*) Δ*argF* *hsdR17 recA1 endA1 gyrA96 thi– 1 relA1*	ATCC PTA-1798

Table S3. Plasmids used in this study.

Plasmids	Characteristic	Reference
pD042	pSW23T::*attC*$_{aadA7}$, *ori*$_{VR6K\gamma}$, *chl*^{R}	[30]
p929	pSU38Δ::*attI1* , *ori*$_{p15A}$, *kan*^{R}	[30]
p3938	pBAD18::*intI1*, *ori*$_{ColE1}$, *car*^{R}	[30]
RP4	IncPα, *car*^{R}, *kan*^{R}	[31]
pBluescript KS^{+}	A plasmid containing *amp*^{R}	Stratagene
pDONR221	A Gateway plasmid containing *attP1*, *attP2*, a suicide gene *ccdB*, and *kan*^{R}	ThermoFisher Cat. No. 2536017
pEXP7-Tet	A linearized Gateway plasmid containing *tet*^{R}, used as a positive control in the *in vitro* recombination	ThermoFisher

Table S4. Medium cultures and selections that were used for bacteria.

Medium	Antibiotics and/or Supplements	Strains & Notes
For functional and mechanistic studies		
MRS broth or agar	–	*L. reuteri*
LB broth or agar	–	*E. coli*

LB broth or agar	–	*S. enterica*
For conjugation and recombination assay.		
LB broth or agar	Chl (25 μg/ml), Kan (25 μg/ml), and DAP (0.3 mM)	β2163 (*pir*$^{+}$ donor)
LB broth or MH agar	Car (100 μg/ml), Kan (25 μg/ml), Glu (10 mg/ml), and dT (0.3 mM)	UB5201 (*pir*$^{-}$ recipient)
LB agar	DAP (0.3 mM), Ara (2 mg/ml), Glu (10 mg/ml), and dT (0.3 mM)	Mix of β2163 & UB5201 (for conjugation and recombination to take place)
LB broth	DAP (0.3 mM), Glu (10 mg/ml), and dT (0.3 mM)	Mix of β2163 & UB5201 (for mixing and diluting)
MHA agar	Chl (25 μg/ml), Car (100 μg/ml), Kan (25 μg/ml), Glu (10 mg/ml), and dT (0.3 mM)	Recombinant
Transformation and recombination assay		
LB broth	Glu (10 mg/ml) and dT (0.3 mM)	UB5201 competent
LB broth	Ara (2 mg/ml), Glu (10 mg/ml), and dT (0.3 mM)	UB5201 (for cell recovery)
MH agar	Car (100 μg/ml), Kan (25 μg/ml), Glu (10 mg/ml), and dT (0.3 mM)	UB5201 (*pir*$^{-}$ recipient)

MH agar	Chl (25 μg/ml), Car (100 μg/ml), Kan (25 μg/ml), Glu (10 mg/ml), and dT (0.3 mM)	Recombinant
For conjugation assay		
LB broth or agar	Car (100 μg/ml) and Kan (25 μg/ml)	BM21
LB broth or agar	Tet (15 μg/ml) and DAP (0.3 mM)	MG1656
LB broth		BM21 (for washing)
LB broth	DAP (0.3 mM)	MG1656 (for washing)
LB agar	Car (100 μg/m1), Kan (25 μg/ml), Tet (15 μg/ml), and DAP (0.3 mM)	Transconjugant
For *in vitro* recombination assay in Gateway reactions		
LB broth or agar		DH5α competent
LB agar	Amp (100 μg/ml)	DH5α recombinant (*attB*-flanked PCR products (*amp*^{R}) & pDONR221)
LB agar	Tet (20 μg/ml)	DH5α recombinant (pEXP7-Tet & pDONR221)

Table S5. Primers used in this study.

Name	Sequences (5'–3')	Purpose	PCR products

			(bp)
16SPCRF	TCGTCGGCAGCGTCAGATGTGTATAAGAGACAGCCTACGGGNGGCWGCAG	Amplifying the V3 - V4 hypervariable sequencing regions	300
16SPCRR	GTCTCGTGGGCTCGGAGATGTGTATAAGAGACAGGACTACHVGGGTATCTAATCC		
16SLR1	GGCTATCACTCTGGGATGGA	Identifying *L. reuteri*	280
16SLR2	GGATACCGTCACTGCGTAAC		
tuf F421	GAA ATC GTTGGTTTG ACTGAAG	Identifying *L. oris*	159
tuf R579	TTCAGCAAGAACTTG ACC A		
yaiOF	TGATTTCCGTGCGTCTGAATG	Identifying *E. coli*	115
yaiOR	ATGCTGCCGTAGCGTGTTTC		
HisJF	ACTGGCGTTATCCCTTTCTCTGGTG	Identifying *S. enterica*	495
HisJR	ATGTTGTCCTGCCCCTGGTAAGAGA		
SWbeg	CCGTCACAGGTATTTATTCGGCG	Validating the *attC* × *attI* recombination	488
MFD	CGCCAGGGTTTTCCCAGTCAC		
12attB1	AAAAAGCAGGCTGGTCGGGGAAATGTGC	Amplifying *attB*-flanked PCR products	1049
12attB2	AGAAAGCTGGGTGGGTCTGAC		

	AGTTACCA	(*amp*^{R} and its promotor)	
attB1 adapter	GGGGACAAGTTTGTACAAAAAAGCAGGCT		
attB2 adapter	GGGGACCACTTTGTACAAGAAAGCTGGGT		
M13	GTAAAACGACGGCCAGT	Validating the *attB* × *attP* recombination	288
HT1	AGGGAATAAGGGCGACACGG		

SUPPLEMENTARY FIGURES

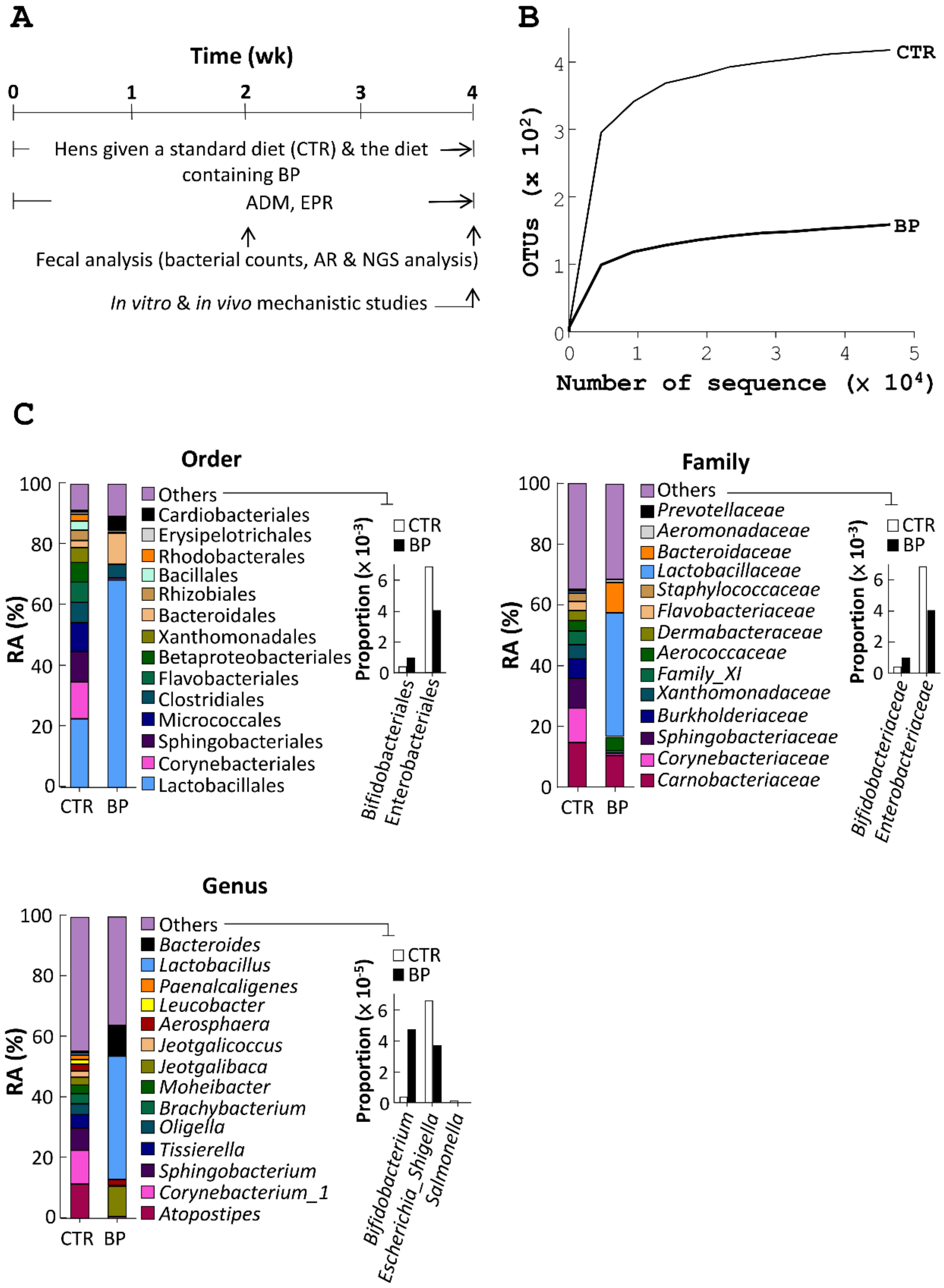

Fig. S1. Experimental design and analysis of 16S rDNA sequencing analysis of gut microbiota in aged hens. (A) A flow chart of the experimental design used in this study. (B) Rarefaction curves of bacterial OTUs in the fecal bacteria of control (CTR) and BP-fed hens (Fig. 2) using the 16S rDNA NGS analysis. (C) Analysis of the relative abundance (RA) (left) of fecal bacteria in (B) and small proportions (right) of fecal bacteria of interest at the level of order, family, and genera.

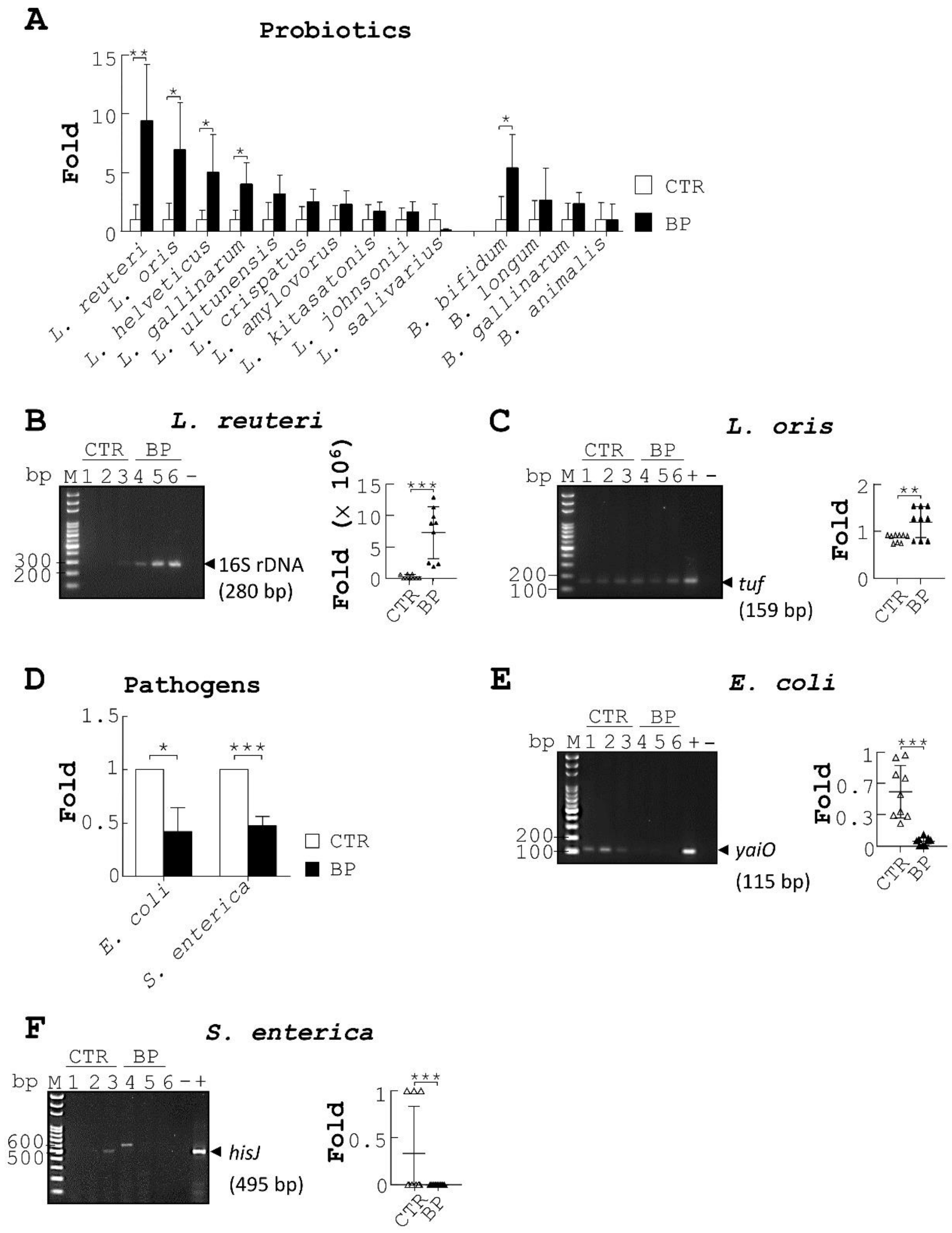

Fig. S2. A proportion of probiotic and pathogenic species in the feces of 58-week-old laying hens. (A) The top 10 *Lactobacillus* species (left) and top 4 *Bifidobacterium* species (right) in the fecal bacteria of control (CTR) and BP-fed hens (Fig. 2) were selected and analyzed based on their fold change using the shotgun metagenomic NGS analysis. (B - C) *L. reuteri* (B) and *L. oris* (C) in the fecal bacteria (A) were

confirmed using PCR with a pair of specific primers for 16S rRNA and *tuf* genes, respectively. Their DNA gels were photographed (left) and their signals in PCR gels were quantified and re-plotted into histograms (right). (D) Fold change of *E. coli* and *S. enterica* in the fecal bacteria of control (CTR) and BP-fed hens (A) were analyzed based on their fold change using shotgun metagenomic NGS analysis. (E - F) *E. coli* (E) and *S. enterica* (F) in the fecal bacteria (A) was detected using PCR with specific primers for *yaiO* and *hisJ*, respectively. Their DNA gels were photographed (left) and their signals in PCR gels were quantified and re-plotted into histograms (right). Data of 3 to 6 repeats (A and B) and 9 repeats (C - E) are presented as the mean ± SD. One-way ANOVA test was used for statistical analysis of differences between groups and P (*) < 0.05, P (**) < 0.01, and P (***) < 0.001 are considered statistically significant.

A

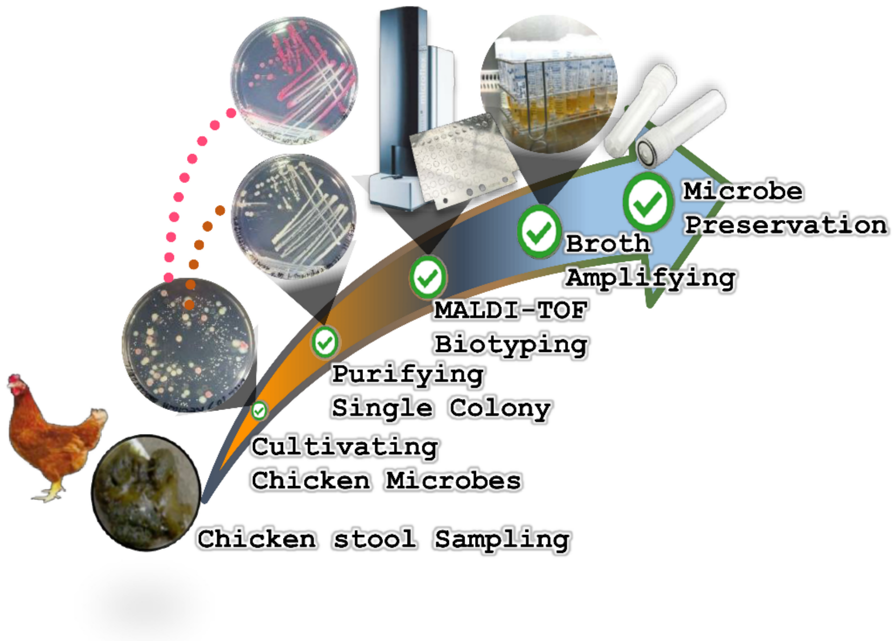

B

No.	Analyte Name	Organism (best match)	Reference	Score Value
01	200918_0_H12	*Lactobacillus reuteri*	DSM 20016T	2.308
02	200918_0_L2	*Lactobacillus reuteri*	DSM 20016T	2.168
03	200918_0_B11	*Lactobacillus reuteri*	DSM 20016T	2.018

No.	Analyte Name	Organism (best match)	Reference	Score Value
01	200918_0_E7	*Escherichia coli*	ATCC 25922 CHB	2.188
02	200918_0_E10	*Escherichia coli*	ATCC 25922 CHB	2.188
03	200918_0_N1	*Escherichia coli*	ATCC 25922 CHB	2.148

No.	Analyte Name	Organism (best match)	Reference	Score Value
01	2_F24	*Salmonella enterica*	Enteritidis 25089078 (PX) MLD	2.251
02	2_P19	*Salmonella enterica*	Enteritidis 25089078 (PX) MLD	2.146
03	2_P10	*Salmonella enterica*	Enteritidis 25089078 (PX) MLD	2.127

Fig. S3. Isolation, selection and identification of probiotics and pathogenic bacteria from chicken stools. (A) A procedure of chicken fecal bacterial collection, cultivation, isolation, MALDI-TOF analysis, and preservation. (B) Characterization of *L. reuteri*, *E. coli*, and *S. enterica* using the MALDI-TOF MS analysis. The spectral profiles of three species were compared with an in-house microbial protein profile database containing > 4000 bacterial species. Top three mostly matched data

of *L. reuteri* (top), *E. coli* (middle), and *S. enterica* (bottom) are indicated.

A

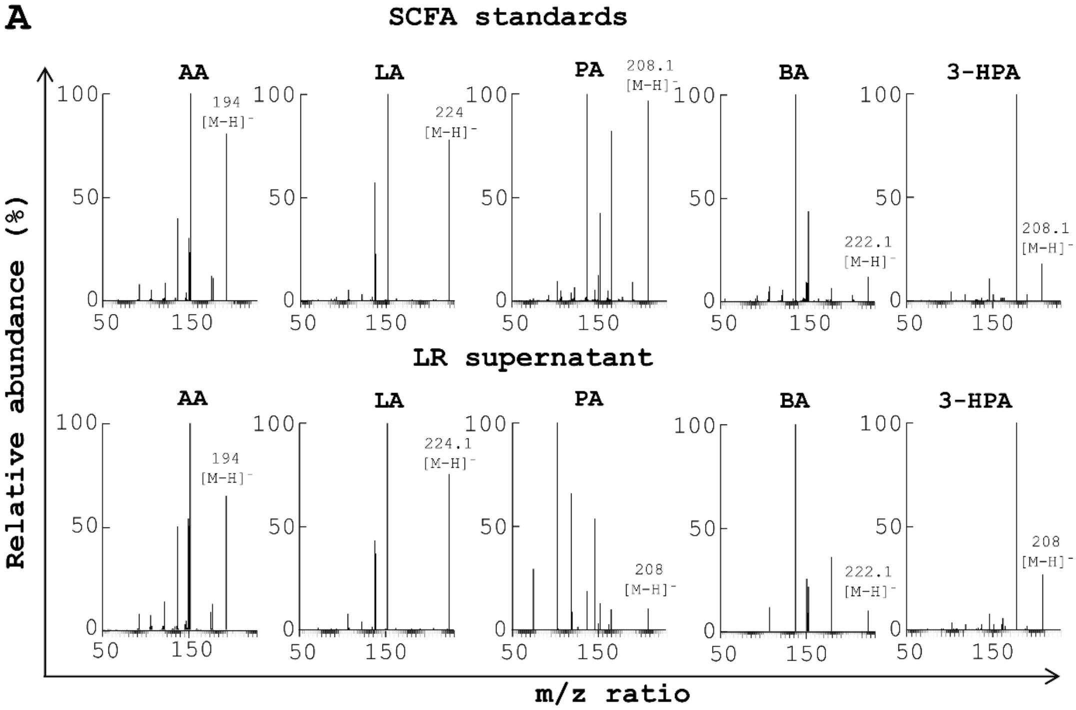

B

Time (h)	Group	SCFA (× 10^{-3} mg/ml)				
		AA	LA	PA	BA	3-HPA
5	CTR	3134.1 ± 151.5	476 ± 2.1	2.9 ± 0.1	5 ± 0.37	497.74 ± 4.53
	BP	3544 ± 43.7*	530.6 ± 8.8**	3.4 ± 0.1*	5.98 ± 0.05*	516.77 ± 18.09
8	CTR	3559.9 ± 39.6	1408.4 ± 16.4	3.9	6.46 ± 0.05	523.20 ± 13.67
	BP	3989.2 ± 19.1***	1518.7 ± 6.9***	4.6 ± 0.1***	7.2 ± 0.1***	747.8 ± 7.1***
16	CTR	3034.9 ± 114.2	1369.8 ± 125.4	1.9 ± 0.2	3.5 ± 0.3	11.9 ± 0.5
	BP	2934.2 ± 141.4	4768.8 ± 106.9***	1.5 ± 0.2	4.6 ± 0.3*	35.4 ± 1.5***

Fig. S4. Identification and quantification of SCFA produced by *L. reuteri*. (A) The 5 SCFA in the supernatant of *L. reuteri* grown in MRS broth (bottom) as well as standard compounds (top), including AA, BA, LA, PA, and 3-HPA, were subjected to LC-MS/MS. Representative MS1 profiles of th_e 5 ion signals corresponding to the 5 SCFA are indicated. (B) The level of the 5 SCFA in the supernatant of *L. reuteri*, grown in MRS broth containing 0.1% methanol (CTR) and BP (4 μg/ml), for the indicated time was analyzed and quantified based on the signal of MS2 in the LC-MS/MS data. Data of 3 repeats are presented as the mean ± SD. One-way ANOVA test was used for statistical analysis of differences between groups and *P*

(*) < 0.05, *P* (**) < 0.01, and *P* (***) < 0.001 are considered statistically significant.

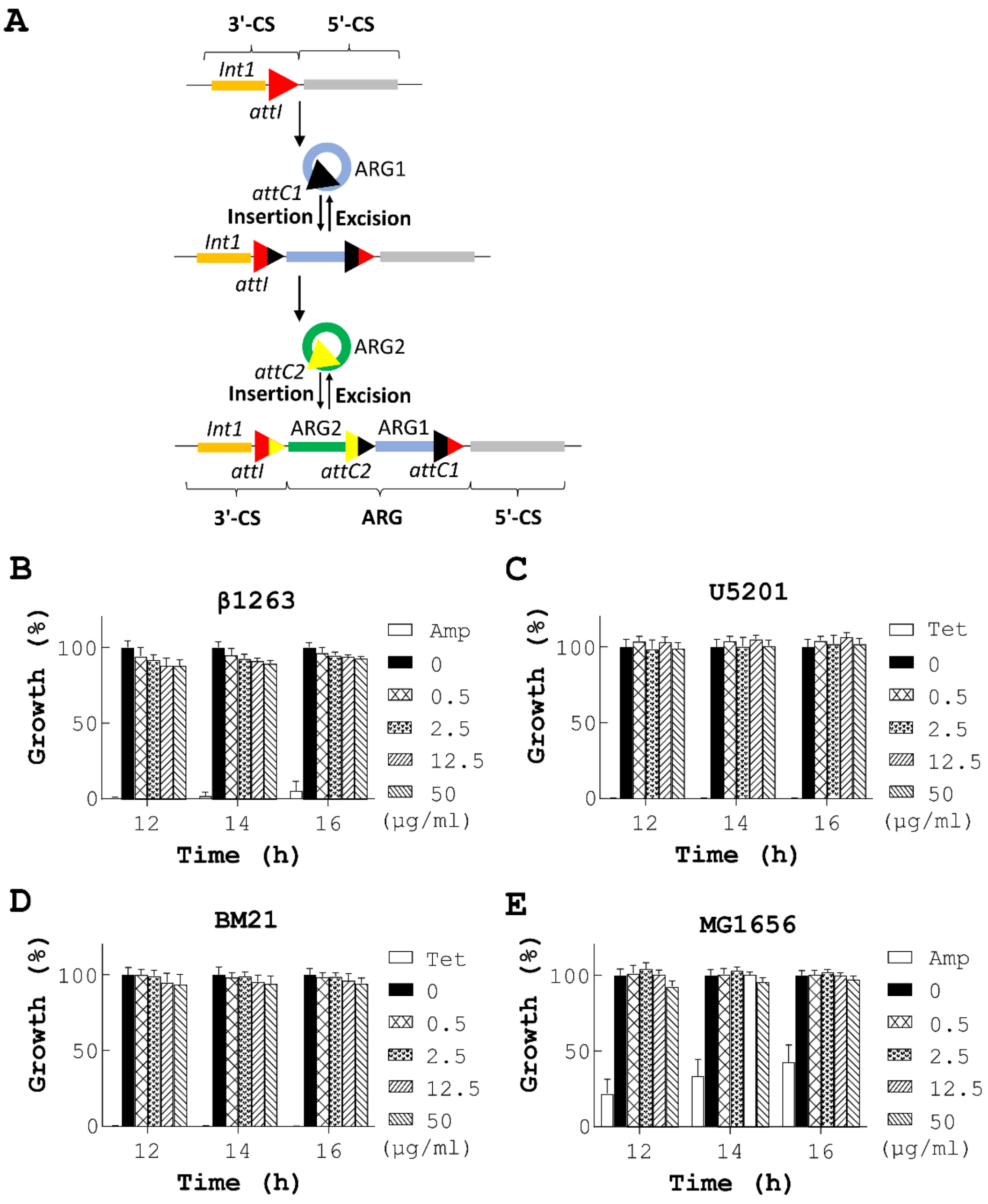

Fig. S5. The structure of the class 1 integron and viability test of bacteria used in Fig. 5. (A) A general structure of class 1 integron and the scheme of ARG cassette insertion or excision. The class 1 integron consists of a 5′-conserved segment (5′-CS), comprising *attC* sites and an integrase gene (*Int1*), ARG cassettes, and a 3′-conserved segment (3′-CS). ARG from integrons, plasmids, and chromosomes can insert into or excise from integron via catalysis of integrase and *att* sites. (B - E) *E. coli* strains,

β1263 (B), U5201 (C), BM21 (D), and MG1656 (E), were grown in LB medium containing a positive control, Amp or Tet, and BP at the indicated dosages for 18 h. The growth rate (%) is indicated. Data of 3 repeats are presented as the mean ± SD. One-way ANOVA test was used for statistical analysis of differences between groups and P (*) < 0.05, P (**) < 0.01, and P (***) < 0.001 are considered statistically significant.
